# A Novel Approach to Evaluating Crosstalk for Near-Infrared Spectrometers

**DOI:** 10.3390/s24030990

**Published:** 2024-02-03

**Authors:** Zemeng Chen, Xinliang Cao, Xianglin Li, Boan Pan, Pengbo Wang, Ting Li

**Affiliations:** 1 Biomedical Engineering Institute, Chinese Academy of Medical Science and Peking Union Medical College, Tianjin 300192, China; b2022015010@student.pumc.edu.cn (Z.C.);; 2 School of Electromechanical and Architectural Engineering, Jianghan University, Wuhan 430056, China; 3 State Key Laboratory of Electronic Thin Films & Integrated Devices, University of Electronic Science & Technology of China, Chengdu 611731, China

**Keywords:** near-infrared spectroscopy, crosstalk analysis, multi-channel, multi-parameter, multi-wavelength

## Abstract

Multi-channel and multi-parameter near-infrared spectroscopy (NIRS) has gradually become a new research direction and hot spot due to its ability to provide real-time, continuous, comprehensive indicators of multiple parameters. However, multi-channel and multi-parameter detection may lead to crosstalk between signals. There is still a lack of benchmarks for the evaluation of the reliability, sensitivity, stability and response consistency of the NIRS instruments. In this study, a set of test methods (a human blood model test, ink drop test, multi-channel crosstalk test and multi-parameter crosstalk test) for analyzing crosstalk and verifying the reliability of NIRS was conducted to test experimental verification on a multi-channel (8-channel), multi-parameter (4-parameter) NIRS instrument independently developed by our team. Results show that these tests can be used to analyze the signal crosstalk and verify the reliability, sensitivity, stability and response consistency of the NIRS instrument. This study contributes to the establishment of benchmarks for the NIRS instrument crosstalk and reliability testing. These novel tests have the potential to become the benchmark for NIRS instrument reliability testing.

## 1. Introduction

Near-infrared spectroscopy (NIRS) has become a valuable tool as a noninvasive analytical technique in the field of human–computer interfaces, medical diagnosis and treatment in recent decades [1,2,3,4]. Near-infrared light can be transmitted to the deep layers of human tissues. NIRS detects changes in multiple physiological parameters in the human body by detecting the optical density of the tissues [5,6]. It has several advantages, including the use of low-cost, silent, transportable/portable instrumentation, and the ability to move freely during the measurements [7,8,9]. Multi-channel and multi-wavelength NIRS is gradually being widely developed and applied due to its advantage of providing more signals and information than other comparable techniques [10,11,12,13,14,15,16,17].

Meanwhile, the crosstalk problem of multi-channel and multi-wavelength NIRS instruments has drawn increasingly more attention, which has led to many hardware design and algorithm solutions aiming to reduce NIRS crosstalk. Crosstalk between channels or wavelengths of NIRS instruments can be effectively reduced by appropriately designing the layout of the printed circuit board (PCB) [18,19]. The essence of crosstalk is a type of energy transfer between adjacent traces. The degree of crosstalk is inversely proportional to the line spacing. In order to improve signal quality and reduce crosstalk between signals during wiring, the distance between traces should be widened as much as possible during design [20,21,22,23]. When drawing PCB lines, 45-degree fold lines should be used, and sharp angles and 90-degree fold lines should be avoided. Folding can reduce the radiation of high-frequency signals. Critical transmission lines should be short and thick. The wiring of adjacent layers should be as vertical as possible. Coupling is easy to achieve when the wires are parallel. When dealing with clock circuit wiring, the wiring from the clock output to the clock input of the device should be as straight and short as possible. Parasitic parameters caused by long wiring will affect the output waveform [24,25]. Reasonable layout and wiring make the instrument highly reliable. Regarding the algorithm solutions to reduce NIRS crosstalk, the existing blood oxygen parameters are calculated from the original data through the Beer–Lambert law formula and some parameters in this process, such as the differential pathlength factor (DPF) [26,27]. DPF is not a definite value. Since DPF estimation requires photon time-of-flight information [28], DPF is first assumed in continuous wave NIRS. Importantly, errors in DPF spectra can cause hemoglobin crosstalk [29], which is detrimental to fNIRS. Some studies suggest relying on multi-range high-density measurements to estimate subject-specific DPF spectral dependence [30]. This reduces hemoglobin crosstalk in high-density recordings.

As the physiological signal of human tissue is very weak, the monitoring system needs high sensitivity and minimization of interference. Crosstalk is an important part of the interference signal. In previous reports [31,32,33] on a NIRS instrument independently developed by the team, the test standards for crosstalk, sensitivity and reliability were different, and the test indicators and schemes were not unified. This constitutes a hindrance to article review and significantly reduces the paper’s credibility, impact and reproducibility. Therefore, it is necessary to develop a set of tests for evaluating the sensitivity, stability and reliability of NIRS instruments.

In this study, the human-blood model test, ink drop test, multi-channel crosstalk and multi-parameter crosstalk test were introduced. An 8-channel, 4-wavelength NIRS instrument developed by our team was used to demonstrate the details and results of the set of tests. The results showed the degree of reliability, sensitivity, stability and response consistency of the test instrument. This study contributes to the establishment of benchmarks for NIRS instrument crosstalk and reliability testing and itself has the potential to become the benchmark for NIRS instrument reliability testing.

## 2. Materials and Methods

**Figure 1:**
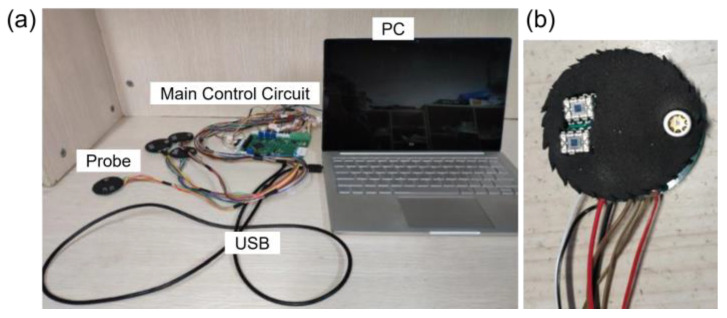
A custom NIRS device used to demonstrate the complete crosstalk test program. (**a**) A photograph of the light probe. (**b**) A photograph of the test equipment.

The human-blood model test, ink drop test, multi-channel crosstalk test and multi-parameter crosstalk test were designed to evaluate the reliability of the test instrument. Here, a functional multi-channel (8 channels), multi-wavelength (4 wavelengths: 760 nm, 850 nm, 910 nm, 970 nm) near-infrared spectrometer developed by our team was employed for the demonstration of a complete crosstalk test program (Figure 1). MATLAB (R2014a, MathWorks, Natick, MA, USA) was employed to analyze the data.

### 2.1. Human-Blood Model Test

In the human-blood model test, the photons emitted by the near-infrared LEDs change in different concentrations of absorbent solutions, causing corresponding changes in the data collected, providing a means to test the reliability, sensitivity, stability and response consistency of a system. In a previous study, several materials were used to simulate the light-absorptive properties of the human body. A number of substances that mimic the absorption of light by the human body are listed and compared in Table 1. Due to the differences in the structures of oxygenated hemoglobin and deoxygenated hemoglobin in different species, this study used human blood to conduct a human-blood model test to verify the sensitivity of NIRS equipment. At the same time, because human blood is difficult to obtain and valuable, this study only conducted experimental demonstrations using a light source with a wavelength of 850 nm for the test equipment.

**Figure 2:**
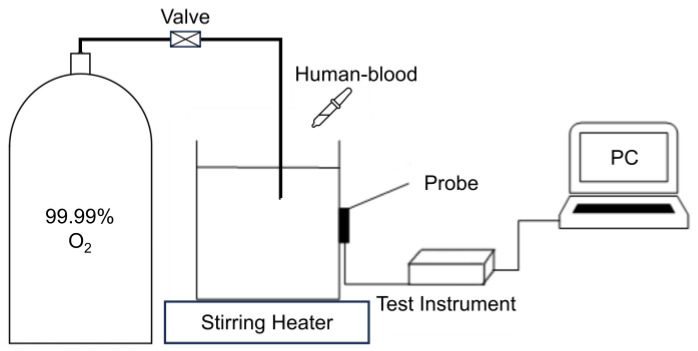
A schematic diagram of the human-blood model test.

As shown in Figure 2, the instrument in the human-blood model test consisted of a polyethylene container, a probe (a light source and a detector), a function module and a computer.

Polyethylene containers and body tissues have the same optical properties. A 22 mm-thick polyethylene cup was placed within a cylindrical polyethylene vessel (CPV) 8.9 cm in diameter. A mixture consisting of 1% Intralipid solution suspended in 450 mL phosphate-buffered saline (PBS) was added to the polyethylene containers. We kneaded 5 g of yeast into the solution before the experiment. The probe of the test equipment used a wavelength of 850 nm, and one photosensitive detector was attached to the polyethylene bag through a window made on the wall of the container. A magnetic stirrer was placed in the solution. After 20 s of baseline measurement, 0.5 mL of human blood was added to the solution. The blood was deoxygenated by yeast during the deoxygenation cycle, and 99.99% oxygen gas was introduced into the solution to oxygenate the blood during the oxygenation cycle. After the blood in the solution was fully oxygenated, the bubbling of 99.99% oxygen was stopped. Then, another cycle of deoxygenation caused by yeast started. During the blood experiment, the solution temperature was kept at 37 °C by a homo-thermal stirring heater.

### 2.2. Ink Drop Test

As shown in Table 1, considering the availability of materials and their stability during the experiment, ink was chosen as the simulated light-absorbing substance in this study.

The photons emitted by the near-infrared LEDs in the ink drop experiments changed accordingly in different concentrations of absorbent solutions, causing corresponding changes in the data collected. In order to test the reliability, sensitivity, stability and response consistency of the equipment, a magnetic stirrer (with rotor and polyethylene stirring container) was used to mix pure black ink and pure water to obtain different concentrations of ink. Two 2.5 mL sterile injection syringes were used to drop small, gradual drops of pure black ink into water. Black blackout sponge and black textile tape were used to prevent light interference from the environment. A 1000 mL large beaker and a 200 mL small beaker were used to hold the solutions during the whole experiment. In addition, since this test is sensitive to light, the influence of ambient light on the experiment should be minimized as much as possible. In this paper, an optical darkroom was chosen as the experimental setting, with all the light sources turned off.

**Figure 3:**
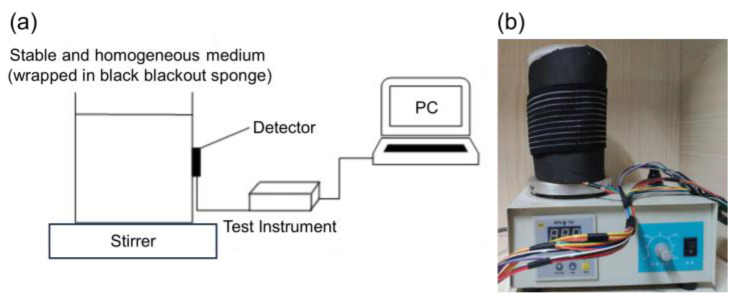
The ink drop test. (**a**) A diagram of the ink drop test. (**b**) A scene photograph of the ink drop test.

Figure 3 shows the diagram and scene photograph of the ink drop test. The experimental procedure was as follows: First, the first probe of the test device was wrapped with a black light-blocking sponge around a polyethylene container with 600 mL of fresh water, ensuring that the highest point of the probe was below the surface of the water. The temperature of the magnetic stirrer was set to a constant 31 °C. The container was placed on the magnetic stirrer tray and the stirring rotor was placed inside the vessel. Then, the materials used in the experiment were prepared: 600 mL of water was added to the small beaker, followed by 1 mL of carbon ink injected into the beaker using a sterile syringe. It was stirred well using a glass rod to obtain a 600-fold diluted solution of black ink because the gradual change of the collected data could not be observed if the concentration of the absorbent dripped into the water was too high. Finally, the device was connected to a computer, the control page of the host computer was opened, and the software parameters such as the serial frequency were set. The device was then turned on and 1 mL of diluted carbon ink was injected with a syringe into the polyethylene container at 1-min intervals. During the whole process of the experiment, the working magnetic stirrer drove the magnetic stirring rotor in the polyethylene container to perform a high-speed rotation and stirring of the liquid so that the drops of diluted carbon ink were fully dispersed in the water; the whole process should be as gentle as possible to avoid the liquid fluctuations causing large fluctuations in the detection data. The computer display of the data curve changes was observed until the end of the experiment. The whole experimental process lasted about ten minutes in total. The variation curves of light intensity at different wavelengths for the first channel of the test instrument and the graph of the liner result of the ink drop test were then plotted.

### 2.3. Multi-Channel Crosstalk Test

For instruments with multiple channels, crosstalk between the channels is inevitable. This part of the experiment was aimed at testing it.

The principle of the channel crosstalk test experiment was to test the crosstalk suffered by each channel in the normal operation of other channels. The specific experimental operation was to use a black opaque sponge to cover the photosensitive detector 1 and place a heavy object on it to ensure that the light would not enter detector 1 outside the instrument while the rest of the detectors were working as normal. The crosstalk of each channel was calculated in turn. In the test, a laser could be chosen as a strong light source to periodically irradiate detector 1. However, in consideration of the availability of materials and the feasibility of the experiment, a smartphone flash was chosen instead as the strong light source. The frequency of periodically turning on and off the flash was set to 1 Hz, and the whole experiment was carried out in an optical dark room to avoid the interference of ambient light.

### 2.4. Multi-Parameter Crosstalk Test

Compared with single-parameter instruments, multi-parameter instruments can obtain richer data and more information. Usually, in order to carry out multiple parameter measurements, it is necessary to integrate several different wavelengths of near-infrared light into the probe, and the crosstalk between parameters is essentially the influence of these different wavelengths of near-infrared light. In order to test the interference between multiple different wavelengths of near-infrared light of the instrument, a multi-parameter crosstalk test was designed in this study.

**Figure 4:**
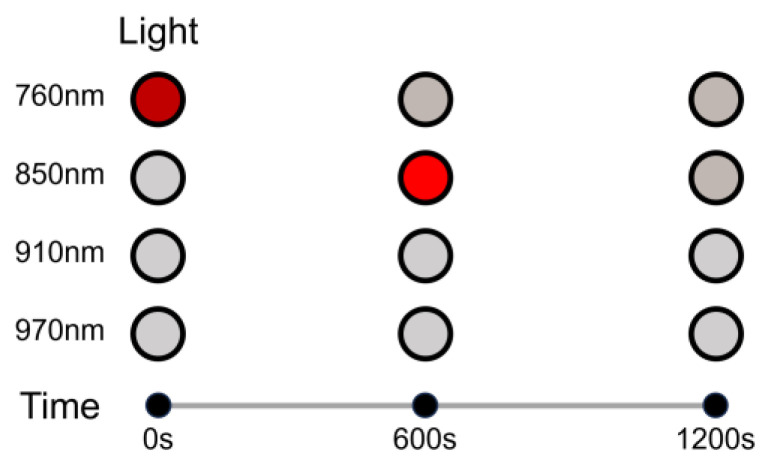
A diagram of the multi-parameter crosstalk test (The circles in red represent light on; the circles in gray represent light off).

In the experimental process, we added ink to water to simulate light passing through a stable environment. First, a polyethylene container was prepared, and then a certain amount of pure black carbon ink was added to the water and mixed well. After that, one of the probes was wrapped with a black shading cloth around the container; we ensured that the whole probe was below the surface of the water. We set the test equipment to light up only at 760 nm wavelength near-infrared light, let it light up for ten minutes (600 s), and then turned off the 760 nm near-infrared light. Then we turned on the 850 nm wavelength near-infrared light and also let it light up for another ten minutes (600 s). Subsequently (as shown in Figure 4), after the four wavelengths of near-infrared light were individually lit for ten minutes, we let the test instrument control the four near-infrared light wavelengths in accordance with the standard method of light collection, that is, time-sharing lighting, at the same time as the main control circuit of the photoelectric detector was set to detect the data collection.

## 3. Results

### 3.1. Human-Blood Model Test Result

**Figure 5:**
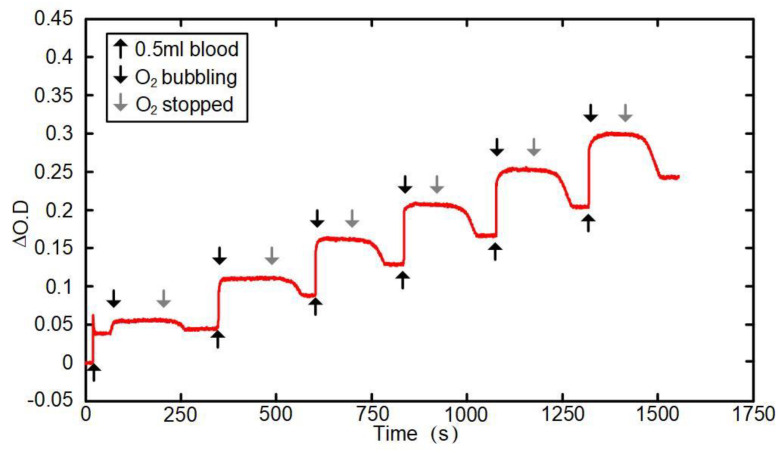
A graph of the human-blood model test.

Figure 5 shows the result of traces obtained in the human-blood model test. The red curve represents the detection results (in ΔO.D value) of the test equipment detector under the 850 nm light source. The upward black arrow represents the moment when 0.5 mL of human blood is dripped, the downward black arrow represents the moment when oxygen pumping starts, and the downward gray arrow represents the moment when oxygen pumping stops. During the first 20 s of the experiment, the ΔO.D value remained near the baseline. After each drop of 0.5 mL of human blood, the ΔO.D value increased significantly until it remained stable after oxygen was pumped in. However, the ΔO.D value decreased to a certain extent after the pumping of oxygen was stopped. The above rules were true for each cycle.

### 3.2. Ink Drop Test Result

**Figure 6:**
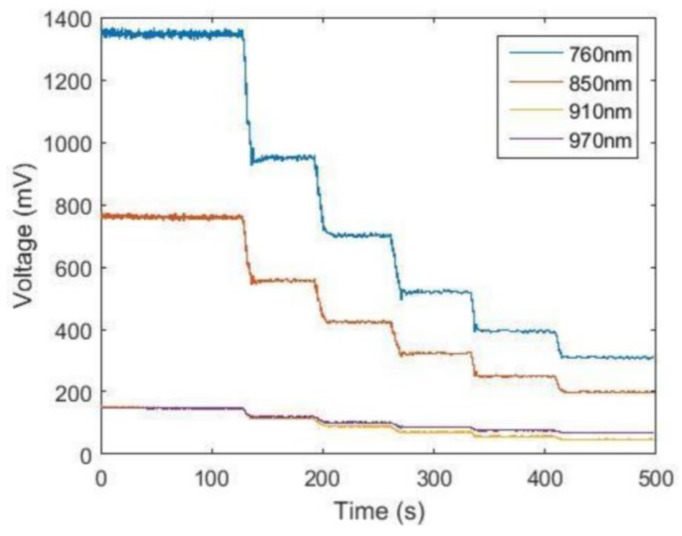
**A** graph of the variation of light intensity at different wavelengths.

The variation curves of light intensity at different wavelengths for the first channel of the test instrument in the test are shown in Figure 6. It can be seen from the figure that, as the amount of ink added increases, the concentration of black ink in the polyethylene container initially filled with water increases, and the high-speed rotation of the rotor causes the ink to be dispersed uniformly at the moment of dropping into the container.

**Figure 7:**
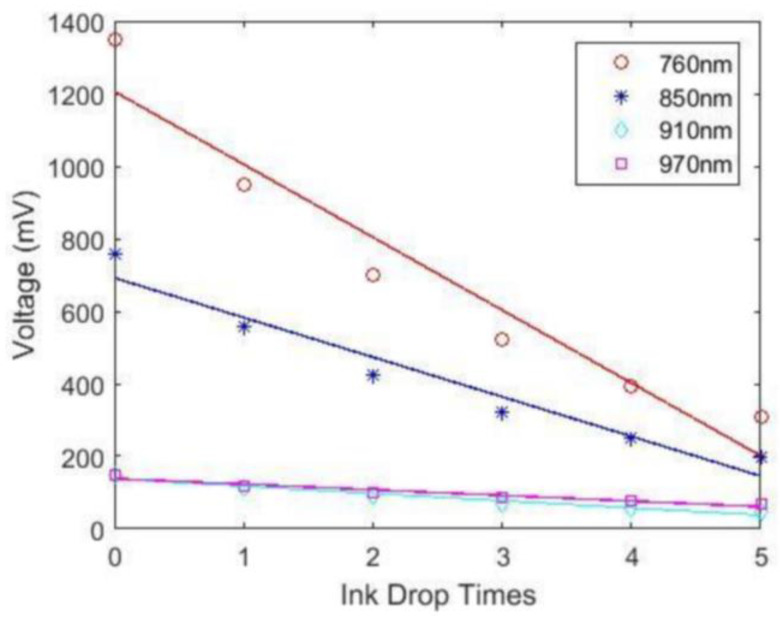
A graph of the linear result of the ink drop experiment on the test equipment.

As shown in Figure 7, the light intensity detected by the photosensitive detector and the number of drops of ink in the solution under test show a linear relationship, indicating that the test instrument has good linearity.

### 3.3. Multi-Channel Crosstalk Test Result

**Figure 8:**
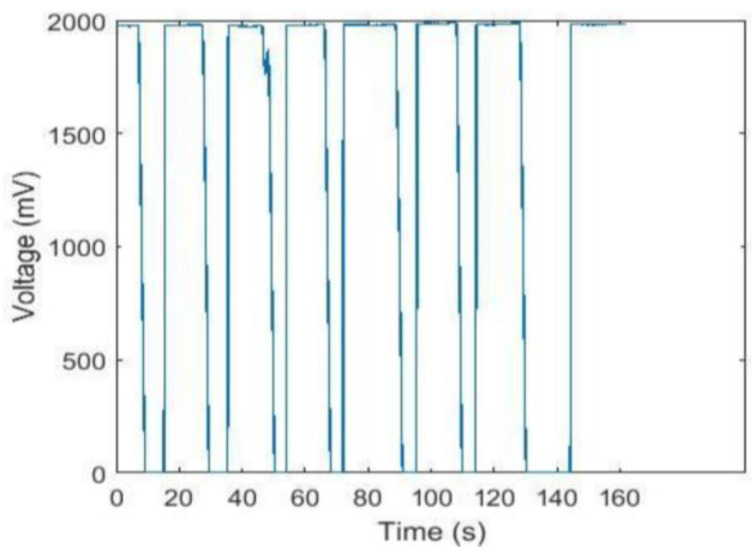
Raw data for channel 2.

**Figure 9:**
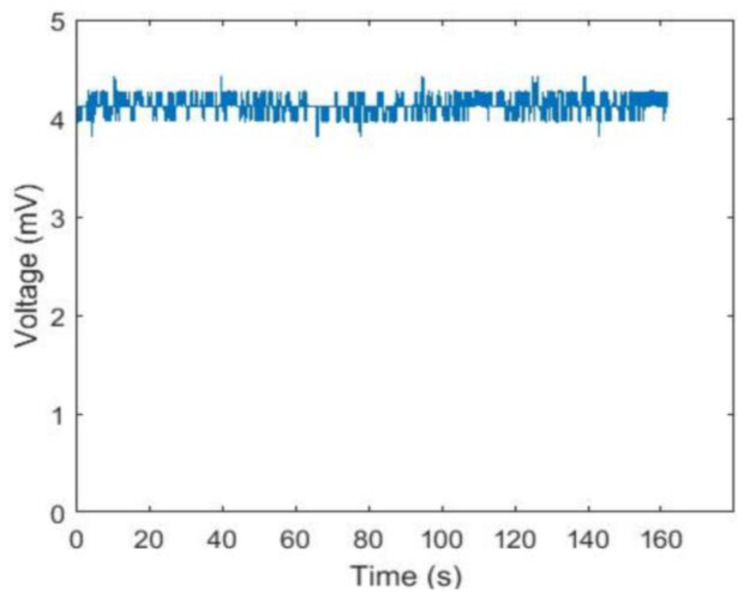
Raw data for channel 1.

The raw channel 2 data are shown in Figure 8. During the experiment, since the excitation to photodetector 2 was a periodic light signal, its response was also a periodic voltage signal. At the same time, all channels except channel 1 were able to detect light from the light source. The raw data of channel 1 is shown in Figure 9. The response of channel 1 was always a very small voltage signal, which was not only interfered with by other channels but also by the dark noise generated by the system itself.

In order to investigate the noise condition of each of the remaining channels when other channels are working normally, the remaining seven channels were also measured according to the above scheme. Table 2 shows the mean measurement (mV) and mean square deviation (mV) results for these eight channels.

### 3.4. Multi-Parameter Crosstalk Test Result

**Figure 10:**
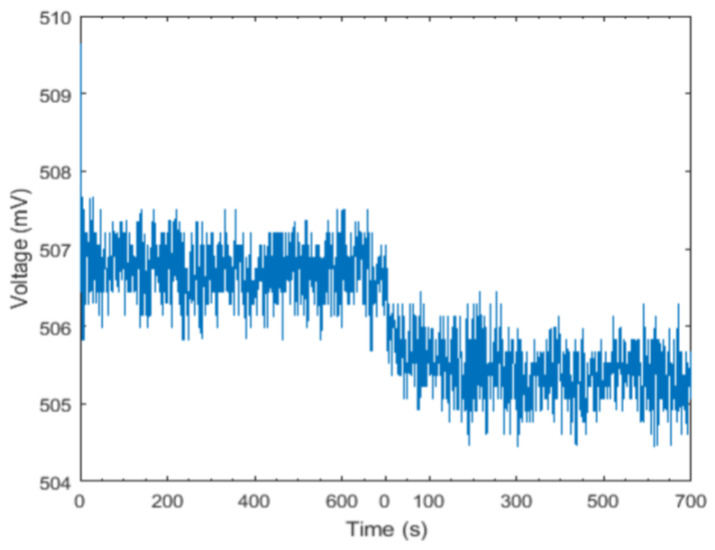
Crosstalk test of 760 nm near-infrared light.

A plot of crosstalk at 760 nm is shown in Figure 10. The first 600 s of the graph is the light intensity detected by the detector when the 760 nm near-infrared light is always lit. The second 700 s is the light intensity of 760 nm near-infrared light detected by the detector when the microcontroller lights up according to the time-lighting mode of near-infrared light in the normal operation of the test instrument.

The crosstalk situation for each wavelength of NIR light is shown in Table 3. Whether in a single-wavelength light or the normal operation of the instrument, when the four wavelengths are lit at different times, the mean square error of the detected results is a very small value.

## 4. Discussion

In this study, an ink drop test, multi-channel crosstalk test and multi-parameter crosstalk test were designed to evaluate the reliability of the instrument, and an 8-channel, 4-wavelength custom device was employed to demonstrate the crosstalk test program.

As shown in Figure 5, during the first 20 s of the experiment, the ΔO.D value remained near the baseline. After each drop of 0.5 mL of human blood, the ΔO.D value increased rapidly until it remained stable after oxygen was pumped in. However, the ΔO.D value rapidly decreased to a certain extent after the pumping of oxygen was stopped. Each cycle followed the above rules. The ability of the ΔO.D value to maintain good stability during both the baseline period and oxygen pumping indicates the reliability of the test equipment. After dripping 0.5 mL of human blood and stopping the oxygen pumping, the ΔO.D value showed a significant sudden increase and decrease, indicating that the sensitivity of the test equipment was good. For any equipment used for testing, if the test results can remain stable when the ratio of oxyhemoglobin and deoxygenated hemoglobin remains consistent, it means that the stability of the equipment is reliable. If the detection results change correspondingly and rapidly when the ratio of oxyhemoglobin and deoxygenated hemoglobin increases or decreases, it means that the sensitivity of the equipment is good.

As shown in Figure 6, the light intensity at the four wavelengths of 760 nm, 850 nm, 910 nm and 970 nm detected by the photosensitive detector decreases in steps according to the same trend of change, and the time point of each change is the moment when the diluted ink is dripped into the container. This shows that the test instrument can reflect the change of absorbent concentration with good sensitivity. If the signals of these four wavelengths between two drops of ink are kept relatively smooth, it indicates that the test instrument has good stability. Broadly speaking, the results of the ink drop experiment show that the more relatively smooth the signals of different wavelengths between the two drops of ink can remain, the better the stability of the test instrument. The Beer–Lambert law indicates that the level of light intensity attenuation is proportional to the concentration of the light-absorbing substance in the solution, since there is a large change in the light intensity signal at the instant the ink is dropped before it stabilizes with stirring. In this paper, the light intensity at each wavelength, when the data are stabilized after each drop of ink, is selected and plotted. As shown in Figure 7, the light intensity detected by the test instrument and the number of drops of ink in the solution under test show a linear relationship, indicating that the test instrument has good linearity, which means it is reliable. The linear relationship between the light intensity detected by the instrument and the number of ink drops in the solution being tested is the same for any instrument. This linear relationship can indicate that the instrument has good linearity and that it is reliable.

According to the raw data for channel 2 and channel 1 in Figure 8 and Figure 9, it can be judged that the performance of the test instrument was excellent. As shown in Figure 8, as the time of the experiment changes, the voltage curve changes very noticeably, which indicates that the detector is very sensitive to detecting the on- or off-state of the corresponding light source. As shown in Figure 9, the response of channel 1 is always a very small voltage signal, which indicates that the test instrument is stable. Simultaneously, its dark noise and the interference value between channels are very small, which indicates that the test instrument is reliable and stable. From Table 2, it can be seen that the mean value of these eight channels is very small, which indicates that these channels will be superimposed on a very small noise signal when the other channels are working normally. These signals have a very small mean square deviation and can therefore be treated as a fixed value. During the monitoring of specific physiological parameters, it is possible to subtract the signal measured by each channel from this superimposed noise signal. The results will be more accurate by subtracting the superimposed noise from the value of the signal measured by each channel during the monitoring of specific physiological parameters. However, in this study, the relationship of distances between sensors and channel crosstalk was not discussed. The design of the probe is critical for the entire monitor hardware design because the arrangement of the light source and photodetector will directly affect the reliability of the signal collected by the probe [34].

It can be seen from Figure 10 that both the first 600 s and the last 700 s of the detector results show very small fluctuations, which also verifies that the instrument has excellent stability in operation. For other equipment, the smaller the voltage fluctuation is when measuring the light of the same wavelength, the better the stability of the instrument. There is an obvious difference between the detector results in the first 600 s and the last 700 s, which indicates that the 760 nm NIR light is interfered with by other wavelengths of NIR light. This is because the difference in detection between these two phases is a very small value compared to the intensity measured by the detector. This indicates that the crosstalk of 760 nm NIR light with other wavelengths is very small and has little effect on actual monitoring results. For other equipment that uses this experiment for crosstalk testing, when the light source emits light of different wavelengths, the more significant the difference detected by the detector, the smaller the crosstalk caused by the light of different wavelengths of the equipment, which indicates better reliability of the equipment. As shown in Table 3, both the mean square error and mean square deviation of the detected results are small values, whether in a single wavelength light or in the normal operation of the instrument, which indicates that the instrument maintains very good stability in both modes of operation. In both modes, the difference in the mean value of the detector results as a percentage of the mean value is very small. This indicates that the four wavelengths of NIR light are not affected by the other wavelengths of NIR light in the normal operation mode and the test instrument is reliable.

## 5. Conclusions

In this study, a set of experiments was designed to evaluate the reliability and crosstalk of the test instrument (a human-blood model test, ink drop test, multi-channel crosstalk test and multi-parameter crosstalk test), and an 8-channel, 4-wavelength custom device was employed for the demonstration of a complete NIRS crosstalk test program. The results demonstrate the reliability, sensitivity, stability and response consistency of the test instrument. As a consequence, this study contributes to the development of industry benchmarking for NIRS equipment. In addition, it has the potential to become the standard for the reliability, sensitivity, stability and response consistency testing of NIRS instruments.

## Figures and Tables

**Table 1:** A comparison of light-absorbing substances that can be added to water.

Absorbent Materials	Advantages	Stability
Whole blood	Providing true tissue spectra and oxygenation capabilities.	Several hours
Fuel molecule	Providing wavelength peak information for spectra.	Several days
Ink	Providing monotonic absorption of the spectrum.	Several weeks

**Table 2:** Crosstalk on each channel.

Channel	Mean (mV)	Mean Square Deviation (mV)
1	4.128	0.104
2	3.981	0.099
3	3.932	0.096
4	3.880	0.098
5	3.964	0.099
6	3.852	0.104
7	4.192	0.103
8	3.992	0.103

**Table 3:** Crosstalk of near-infrared light at various wavelengths.

Wavelength (Single Wavelength Always Lit)	Mean (mV)	Mean Square (mV)	Wavelength (Four Wavelengths Lit Up at Different Times)	Mean (mV)	Mean Square (mV)	Mean Difference	Mean Difference/Mean
760 nm	506.724	0.377	760 nm	505.440	0.385	−1.284	−0.003
850 nm	306.756	0.346	850 nm	306.485	0.364	0.018	5.775 × 10^−5^
910 nm	65.980	0.314	910 nm	66.113	0.323	0.008	1.225 × 10^−4^
970 nm	81.589	0.323	970 nm	81.681	0.331	0.008	1.028 × 10^−4^

## Data Availability

Dataset available on request from the authors. (The data that support the findings of this study are available on request from the corresponding author, [Ting Li, liting@bme.cams.cn], upon reasonable request).
